# Discrimination of Gastrointestinal Nematode Eggs from Crude Fecal Egg Preparations by Inhibitor-Resistant Conventional and Real-Time PCR

**DOI:** 10.1371/journal.pone.0061285

**Published:** 2013-04-19

**Authors:** Janina Demeler, Sabrina Ramünke, Sonja Wolken, Davide Ianiello, Laura Rinaldi, Jean Bosco Gahutu, Giuseppe Cringoli, Georg von Samson-Himmelstjerna, Jürgen Krücken

**Affiliations:** 1 Institute for Parasitology and Tropical Veterinary Medicine, Freie Universität Berlin, Berlin, Germany; 2 Institute for Parasitology, University for Veterinary Medicine, Hannover, Germany; 3 Department of Pathology and Animal Health, Faculty of Veterinary Medicine, University of Napels Frederico II, Naples, Italy; 4 University Teaching Hospital of Butare, Faculty of Medicine, National University of Rwanda, Butare, Rwanda; New England Biolabs, United States of America

## Abstract

Diagnosis of gastrointestinal nematodes relies predominantly on coproscopic methods such as flotation, Kato-Katz, McMaster or FLOTAC. Although FLOTAC allows accurate quantification, many nematode eggs can only be differentiated to genus or family level. Several molecular diagnostic tools discriminating closely related species suffer from high costs for DNA isolation from feces and limited sensitivity since most kits use only small amounts of feces (<1 g). A direct PCR from crude egg preparations was designed for full compatibility with FLOTAC to accurately quantify eggs per gram feces (epg) and determine species composition. Eggs were recovered from the flotation solution and concentrated by sieving. Lysis was achieved by repeated boiling and freezing cycles – only *Trichuris* eggs required additional mechanic disruption. Egg lysates were directly used as template for PCR with Phusion DNA polymerase which is particularly resistant to PCR inhibitors. Qualitative results were obtained with feces of goats, cattle, horses, swine, cats, dogs and mice. The finally established protocol was also compatible with quantitative real-time PCR in the presence of EvaGreen and no PCR inhibition was detectable when extracts were diluted at least fourfold. Sensitivity was comparable to DNA isolation protocols and spiked samples with five epg were reliably detected. For *Toxocara cati* a detection limit below one epg was demonstrated. It was possible to distinguish *T. cati* and *Toxocara canis* using high resolution melt (HRM) analysis, a rapid tool for species identification. In human samples, restriction fragment length polymorphism (RFLP) and HRM analysis were used to discriminate *Necator americanus* and *Ancylostoma duodenale*. The method is able to significantly improve molecular diagnosis of gastrointestinal nematodes by increasing speed and sensitivity while decreasing overall costs. For identification of species or resistance alleles, analysis of PCR products with many different post PCR methods can be used such as RFLP, reverse-line-blot, Sanger sequencing and HRM.

## Introduction

Gastrointestinal nematodes are important pathogens in veterinary and tropical medicine and due to the zoonotic potential of some species (e.g. *Toxocara* spp., *Ascaris suum*, *Ancylostoma caninum*), close collaboration between veterinarians and tropical disease specialists would improve intervention measures. Diagnosis of gastrointestinal nematodes still relies predominantly on coproscopy using microscopic examination. Traditional coproscopic diagnostic tools offer the advantage of being rapid and inexpensive, allowing either sensitive qualitative (*e.g.* simple flotation in tube, ether-concentration methods) or moderately sensitive quantitative (*e.g.* McMaster and Kato-Katz methods) determination of the infection status of humans and animals including e.g. ruminants, equines, cats and dogs. The recently introduced use of FLOTAC chambers [1] now theoretically allows sensitivities as low as one egg per gram feces (epg) to be combined with highly quantitative and accurate data acquisition [2] and has been shown to be superiors to Kato-Katz for detection of human helminthosis [3], [4]. However, due to the fact that many nematode eggs are virtually indistinguishable using conventional microscopy, there is an increasing need to develop new diagnostic tools based on genetic discrimination of these organisms [5], [6], [7]. This is particularly true for the order Strongylida that includes many species frequently found to co-parasitize the same host. However, different strongylid parasite species differ substantially in their pathogenicity and thus require different interpretation of fecal egg counts before treatment decisions and molecular techniques are required to identify the species present.

The amount of DNA found within an individual egg will depend on several factors including the nematode species, the developmental stage that is excreted with the feces, the storage time and conditions (in particular temperature, humidity and availability of oxygen) [8], [9]. Therefore, nematode DNA content in a sample will always vary with several factors that are difficult or impossible to control – at least for routine diagnostics with veterinarians sending in samples to central laboratories. Even more importantly, egg output per female worm differs dramatically between different gastrointestinal nematode genera and/or species with *Haemonchus* and *Chabertia* being highly fecund with egg numbers reflecting worm burden, while members of the Ostertaginae produce only low and extremely variable numbers of eggs. For the latter, the epg is not of any prognostic value for estimation of worm burden even in a mono-specific infection [6]. Therefore, answers to qualitative questions (*e.g.*: Is *Haemonchus* present or not?) or semi-quantitative questions (e.g.: Is the majority of nematode DNA in the sample from a parasite with low or high pathogenicity?) are often sufficient for treatment decisions and would in fact be an important improvement compared to the current state of the art.

Although direct PCR from trichostrongylid eggs manually picked from purified egg suspensions has been previously described to be suitable for genus identification [10], use of this method never became widespread and a reliable amplification directly from eggs obtained by flotation could not be reproduced by another research group [11]. Real-time assays for quantification of trichostrongylids have been published, however, multi-plexing PCRs for several nematodes appeared to be difficult when using related target regions due to suppression of amplification from low abundant targets [12], [13]. Recently published major breakthroughs towards standardized molecular diagnostic tools currently use eggs obtained by flotation followed by several washing steps and DNA extraction with standard commercial purification systems to reliably remove PCR inhibitors present in fecal samples [14], [15]. The future aim in the latter project is to omit egg purification by flotation and instead use direct DNA extraction from feces. For this purpose, large scale DNA isolation from several grams of feces will be required in order to avoid loss of sensitivity. This approach has several obvious advantages such as feasibility for complete automation and easy control of potential cross contamination by using only single-use articles in the laboratory. On the other hand, large scale DNA extraction will significantly contribute to the overall costs and probably this method will be only economic for large laboratories handling at least several hundred samples per month and large farms that are probably more willing to spend money on precise diagnosis than small farms. In particular, cheap diagnostic tools are needed for human samples since many people in underdeveloped countries have no money to spend for sophisticated methods. We were therefore looking for a simple and inexpensive method to specifically identify the most relevant parasite species involved in gastrointestinal infections of humans and animals. To this end it was decided to take advantage of new, inhibitor-resistant thermostable DNA polymerases [16] which have already been shown to be able to overcome inhibition of PCR by components present in blood [17]. We also wanted to combine the newly developed method with quantification of nematode eggs by FLOTAC since we routinely use this method for research purposes in our field studies.

## Materials and Methods

### Ethic Statement

All animal experiments were approved by responsible local administrations and were in accordance with German (Tierschutzgesetz) and European (European directive 2010/63/EU) regulations regarding animal welfare. Experiments with dogs and cats were carried out in Hannover and approved by the Landesamt für Verbraucherschutz und Lebensmittelsicherheit (LAVES) under the reference number 509c-42502-01A38 and experiments with ruminants and horses were performed in Berlin and approved by the Landesamt für Gesundheit und Soziales (LAGeSo) under the reference number L 0088/10. Dogs and cats were bred for research purposes by the Institute for Parasitology (Hannover). Calves were bought from local farmers and kept in the animal house of the Institute for Parasitology and Tropical Veterinary Medicine (Berlin). Swine samples were collected by the farmer’s local veterinarian and sent in for diagnostic purposes. Permission to use the rest of the material for research purposes was given by the owner. The goats were bred and are owned by the Institute for Parasitology and Tropical Veterinary Medicine (Berlin). The horses were bought for research purposes in 2004 and are owned by the Institute for Parasitology and Tropical Veterinary Medicine (Berlin). Goats and horses are permanently kept on a pasture on the university campus.

Human stool samples, positive for hookworm eggs, were obtained within the collaborative research project “HIV infection, malaria, geohelminths and malnutrition among children in Butare, Rwanda” that has been approved by the Rwanda National Ethics Committee under reference number N°136/RNEC/2009. Among the documents reviewed by Rwanda National Ethics Committee and covered by this approval is the Material Transfer Agreement between Butare University Teaching Hospital and the Institute of Tropical Medicine and International Health, Charité-University Medicine, Berlin, Germany. Stool samples were stated among the samples to be transferred for geohelminth studies. The material transfer agreement approved the use of the transferred samples for research purposes. Samples were sent anonymously to Berlin. All children were under five years old. Parents provided informed written consent. Furthermore human samples were kindly collected during a survey on intestinal parasitic infections on the shore of Lake Victoria, Tanzania. (a separate study conducted by DI in collaboration with researchers at San Raffaele Hospital). The overall protocol of the study was reviewed and approved by the Ethic Committee of the Faculty of Medicine, San Raffaele Hospital, Milan, Italy. A separate ethical clearance was obtained from the Bukumbi hospital management board (Mwanza, Tanzania; Bukumbi hospital, Bukumbi, Tanzania; archdiocese of Mwanza) and the chiefs of the villages of Bukumbi, Isamilo, Chole and Kigongo). After providing a specific diagnosis for the patients, samples were anonymized and send to Berlin. The parents or legal guardians of all child participants signed a written informed consent sheet on the behalf of all subjects.

### Source of Feces

All goat and swine feces were obtained from naturally infected animals. Fecal samples from cattle, horses, dogs, and cats were obtained from animals experimentally infected for routine parasite isolate maintenance at the Institute for Parasitology and Tropical Veterinary Medicine of the Freie Universität Berlin (*Ostertagia ostertagi* and *Cooperia oncophora* in cattle and small strongyles in horses) and at the Institute for Parasitology of the University for Veterinary Medicine Hannover (*T. canis*, *Uncinaria stenocephala* and *A. caninum* in dogs and *T. cati* and *Ancylostoma tubaeformae* in cats). *Trichuris muris* and *Trichuris vulpis* positive fecal samples were a gift of Claudia Welz (Bayer Animal Health, Monheim).

The survey in Rwanda was performed among children under five years of age in southern highland Rwanda [18] and the samples were stored frozen at −20°C. Samples had been used for other purposes previously and were thawed for the third time for these experiments. A small aliquot (100 µl) was removed from the sample and processed as detailed below. The human samples collected in Tanzania were conserved in Lugol’s iodine. For this purpose, 10 g fecal matter was mixed with 1% Lugol’s iodine solution (100% Lugol’s iodine is 250 g KI and 125 g I_2_ dissolved in 250 ml water) in a 60 ml screw cap tube which was completely filled and then closed to exclude any air.

### Preparation of Nematode Egg Samples

**Figure 1:**
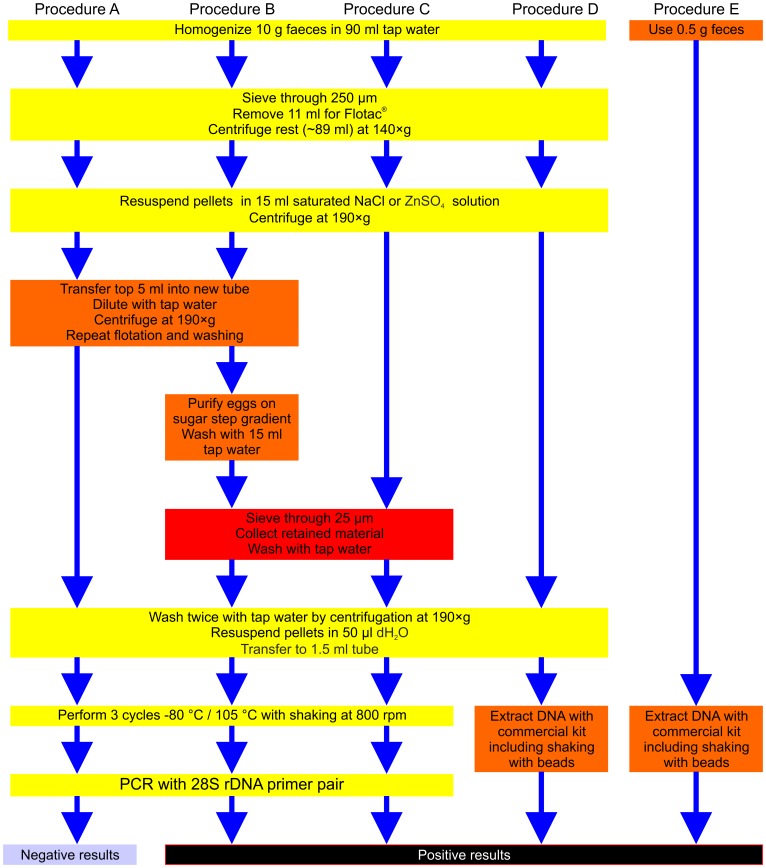
Flow chart comparing different egg purification protocols. Steps that were used in all protocols are shown in yellow, variant purification protocol steps in either orange or red. The latter color indicates mandatory steps for successful amplification in the final protocol in method C. The fourth protocol was modified from [14] by increasing the amount of feces to 10 g to allow comparison with the other approaches.

All samples (except human samples from Rwanda) were analyzed using the standard FLOTAC protocol. Since this protocol uses 10 g of feces but only 11% of the prepared fecal suspension is finally loaded into the counting chamber, there was another 89% that could be used for further molecular analyses. In order to combine the PCR method with the egg quantification using FLOTAC, several different methods to further enrich nematode eggs for molecular analysis were compared. The different procedures are summarized in the process flow diagram shown in Figure 1. For optimization of the new method, the procedures A, B, and C were initially tested with goat feces. Since compatibility with the FLOTAC method was considered a requirement for the method to be developed, nothing was changed regarding the standard FLOTAC quantification of eggs. Therefore, 10 g of fecal samples were homogenized in 90 ml tap water and filtered through a 250 µm stainless steel sieve. Then, 11 ml of the flow through were removed for FLOTAC analysis and 89 ml remained for PCR template preparation. The latter fraction was pelleted at 140×g for 5 min before pellets were re-suspended in 15 ml flotation solution, respectively. Both, saturated NaCl or ZnSO_4_ solution, were tested for compatibility with the new PCR method. These suspensions were either filled in two FLOTAC chambers or in two 50 ml tubes and centrifuged at 190×g for 5 min. Three different approaches for the next steps in PCR template preparation were then compared:

#### Procedure A

The top 5 ml of the supernatant were transferred to a fresh tube, diluted with tap water to 50 ml and centrifuged at 190×g for 5 min. The pellet was subjected to a second round of flotation and two washing steps.

#### Procedure B

The top 5 ml were washed as described in Method A. Additionally eggs were then purified using a standard sucrose step gradient with steps of 10%, 25% and 40% saturated sucrose solution. After centrifugation at 190×g, eggs were collected at the border between the top and middle layers and then applied onto a 25 µm sieve with about 7 cm diameter (precision woven nylon polyamid mesh purchased from Sefar GmbH, Wasserburg/Inn, Germany) to remove the sucrose and small particulate material. The material retained on the sieve was thoroughly washed with double-distilled water and flushed into a 15 ml tube and centrifuged.

#### Procedure C

The top 5 ml of the flotation solution were immediately poured onto the 25 µm sieve without a second NaCl/ZnSO_4_ flotation and without purification via a sugar gradient. After washing, the material retained on the sieve was again collected in a 15 ml tube.

For all three procedures, enriched egg preparations were then centrifuged at 190×g for 5 min, washed twice with sterile, double-distilled water, re-suspended in 100 µl and finally transferred to a 1.5 ml microcentrifuge tube.

For frozen human samples the method was downscaled and no examination with FLOTAC was performed. Briefly, samples were washed through a 250 µm sieve (precision woven nylon polyamid mesh with 1.5 cm diameter) with 50 ml water. After sieving on a 25 µm mesh, the material retained on the sieve was collected and 15 ml saturated NaCl solution added to float the eggs. Samples were centrifuged at 190×g for 5 min and eggs from the top 1 ml were again collected on a 25 µm mesh. The material retained was washed into a microcentrifuge tube, centrifuged at 140×g for 5 min and re-suspended in 10 µl double-distilled water.

Human samples shipped in 1% Lugol’s iodine were treated the same way as normal samples but additional washing steps had to be included in the protocol before the flotation step until the iodine odor was no longer detectable.

After usage, all sieves were thoroughly washed first with tap water, than with distilled water, treated with 3% sodium hypochlorite for at least 3 h (or overnight) and finally washed again with double-distilled water.

### Egg Lysis

In order to achieve a simple, fast, inexpensive and minimally laborious method to lyse nematode eggs, we focused on the evaluation of freeze/boiling to crack the eggs and release sufficient amounts of genomic DNA for qualitative analysis of infection. Three cycles of freezing the eggs rapidly at −80°C followed by incubation in a heating block set at 105°C for 10 min with shaking at 800 rpm were routinely used. For bovine samples, results improved when 5 freeze/boil cycles were used and the method was therefore adapted accordingly for detection of cattle parasites. Eggs of *Trichuris* spp. had to be disrupted mechanically by rapid shaking in the presence of beads (taken from the NucleoSpin® Soil DNA extraction kit, Macherey-Nagel) for 5×1 min with 1 min pausing between shaking intervals using a SpeedMill (Jena Bioscience, Germany). Negative feces controls were prepared from feces of animals not infected with gastrointestinal nematodes.

### Conventional Direct PCR on Crude Egg Preparations (d-PCR)

Primer pairs were designed to amplify small amplicons (<300 bp). All primer sequences together with annealing temperatures and amplicon sizes are listed in Table S1. Pan-nematode specific primers were directed to contain variable fragments of the 28S or 18S rDNA of nematodes. A primer pair aimed to amplify a partial fragment of the ITS-1 of trichostrongylids was designed by combining a primer binding to the end of the 18S rDNA with a slightly degenerated primer targeting a conserved region in the ITS-1 of trichostrongylids. Finally, species-specific primer pairs for important trichostrongylid parasites of ruminants were obtained from the ITS-2 region.

PCR was carried out either with Maxima Hot Start DNA polymerase (Fermentas; only in initial trials) or Phusion Hot Start II High Fidelity DNA polymerase (Finnzymes/Fermentas). For Maxima Hot Start polymerase, reactions contained 2 µl template, 200 µM dNTPs, 0.3 µM of each primer, and 1 U polymerase in 25 µl 1×buffer. PCRs were carried out in a PTC-1000 cycler (Bio-Rad) with 4 min 95°C as initial denaturation, followed by 40 cycles 95°C for 15 s, annealing at the primer specific temperature (Table S1) for 30 s and extension at 72°C for 30 s. Reactions with Phusion DNA polymerase were set up to contain 2 µl of template, 200 µM dNTPs, 0.5 µM of each primer, and 0.4 U polymerase in 20 µl 1×Phusion HF buffer. Here initial denaturation was performed at 98°C for 30 s and followed by 40 PCR cycles with 10 s denaturation at 98°C, 30 s annealing at the primer-specific temperature and 30 s extension at 72°C. As positive controls, freshly diluted plasmid DNA (1 ng) (either the amplicon or a complete ITS-1 or ITS-2 fragment cloned in pCR4 TOPO (Invitrogen) were used.

### Determination of Detection Limits and Analysis

For comparison with the recently described method [14], [15] and determination of detection limit of these methods two additional procedures were evaluated: Procedure D was performed following the methods described by Bott et al, 2009; Procedure E was performed following the manufacturers instruction using the NucleoSpin® Soil DNA extraction kit (Macherey-Nagel).

For this purpose feces (65 g) from dewormed sheep and negative for nematode eggs as determined by FLOTAC were spiked with *C. oncophora* eggs and thoroughly mixed to achieve epgs of 250, 150, 100, 50, 25 and 5. For each egg concentration three replicates were produced. From every fecal sample 20.5 g aliquots were drawn. Then, 0.5 g from each aliquot were used to extract DNA directly from fecal matter with the NucleoSpin® Soil DNA extraction kit (Macherey-Nagel = Procedure E). The kit has recently been reported to be particularly efficient in extraction of DNA from coccidian oocysts from fecal samples [19] which are similar to nematode eggs regarding their tenacity. The first step in the DNA extraction protocol involves mechanical disruption of eggs using vigorous shaking in the presence of beads. The remaining 20 g were used for FLOTAC analysis to obtain the actual epgs. Half of the obtained suspension (89 ml) was used to purify eggs using a protocol similar to [14] but with a higher initial amount of feces (10 g vs. 4 g = Procedure D). After flotation, eggs were diluted and washed twice with tap water by dilution and centrifugation in 50 ml tubes. Then, pellets containing eggs and fecal debris were subjected to DNA extraction using the NucleoSpin® Soil DNA extraction kit (Macherey-Nagel) as described above. The other 89 ml were analyzed using Procedure C. Final volume for all samples was 50 µl.

### Restriction Fragment Length Polymorphism Analysis

PCR product purification, restriction and separation on the Bioanalyzer 2100 (Agilent Technologies) were performed essentially as described recently [20]. Briefly, PCR products (150 ng) were digested in 5 µl with *Rsa*I or *Taa*I (Fermentas) for 3 h. Then, 1 µl of the reaction was separated using the DNA1000 LabChip® kit (Agilent Technologies) according to the manufacturer’s instructions.

### Real-time d-PCR to Determine PCR Efficacies

For real-time and high-resolution melt analysis, EvaGreen (Jena Bioscience) was added to the reaction at a final concentration of 400 µM. Reactions were carried out in a Bio-Rad CFX-96 cycler with cycling conditions for the 28S rDNA primer pair as described above. Bio-Rad CFX manager 2.0 software was used throughout the experiments. Fluorescence was recorded at the end of each extension step. As standards, dilution series of supercoiled plasmids with known copy numbers were freshly prepared.

To evaluate PCR efficacies, serial dilutions of supercoiled plasmid and extracts from *T. cati* positive cat feces obtained using Procedure C were used as template for real-time PCR. Reactions were carried out in quadruplicate. Baseline correction was applied and auto-calculation of thresholds for C_q_ determination was done with default parameters by CFX manager 2.0. Then, C_q_ values were plotted on the ordinate with log_10_ (starting quantity) on the abscissa using GraphPad Prism 5.0. For both datasets nonlinear regression using the GraphPad algorithm “semi-logarithmic curve fit with logarithmic×data” was performed and slopes were compared between both groups. The amplification efficacy was calculated as E = 10^(−1/slope)^. In addition, LinRegPCR 11.0 [21] was used to calculate PCR efficacy from individual PCR reactions. For this purpose, raw data of amplification plots were exported from the CFX manager and imported into LinRegPCR, choosing the Bio-Rad iCycler format for input. PCR efficacy in individual samples was then calculated with a common threshold and individual windows of linearity for all baseline-subtracted samples. A Student’s t test was performed to identify significant differences in PCR efficacy between plasmid and fecal samples.

### Real-time PCR to Estimate Sensitivity and Possible Correlation between epgs and C_q_ Values

Fecal samples from sheep were spiked with defined numbers of *C. oncophora* eggs and subjected to the optimized Procedure C, the isolation of eggs by flotation followed by DNA isolation (Procedure D) and direct DNA isolation from feces (Procedure E). From every extract/DNA-sample, 1 µl was used as template for real-time PCR with the 28S rDNA primer pair in the presence of 400 µM EvaGreen. C_q_ values were plotted against egg numbers as determined by FLOTAC. Regression curves were calculated in GraphPad Prism 5.0 using the “semi-logarithmic curve fit with logarithmic×data” function.

### Compatibility of d-PCR with High-resolution Melt (HRM) Analysis

For evaluation whether d-PCR is compatible with species identification by HRM analyses, fecal extracts (obtained using Procedure C) corresponding to 150 *T. canis*, *T. cati* or a mixture of 75 *T. canis* and 75 *T. cati* eggs per 1 µl were diluted 1∶4 and used as template for real-time PCR in the presence of EvaGreen as described above. At the end of the run, a high-resolution melting curve was recorded by slowly increasing the temperature from 62°C to 98°C with a slope of 0.1°C/10 s. Fluorescence was recorded continuously throughout the melting process. Raw relative fluorescence units (RFU) data and their first derivative were obtained in BioRad CFX manager 2.0. Data were than normalized using BioRad Precision Melt Analysis™ Software V1.0 which was also used to generate difference plots and to assign the different melt curves in clusters. For clustering, a melt curve shape sensitivity of 50% and a Tm difference threshold of 0.15 were used (default parameters).

## Results

### Evaluation of Different Egg Purification Protocols and Thermostable DNA Polymerases

Purification Procedures A–C, as described in materials and methods and outlined in the flow scheme in Figure 1, were initially compared using goat fecal samples. Eggs were lysed before PCR by repeated cycles of freezing and boiling. Figure S1 shows eggs as typically obtained with this method after freezing/boiling lysis. Despite apparently only incomplete lysis of eggs, the amount of DNA released turned out to be sufficient for successful amplification of nematode DNA from samples using conventional PCR if the epg was at least 25. As shown in Figure S1B the amount of fecal debris present in boiled fecal sample extracts is often much higher than that of nematode eggs.

To compare PCR results after different purification procedures, the primer pair for 28S rDNA was used. Simply washing the fecal suspension containing the eggs by dilution and centrifugation (method A) was insufficient to obtain a PCR product (data not shown). In contrast we were able to amplify the target sequence using Phusion II DNA polymerase by concentration and purification of eggs on a step sucrose gradient followed by sieving (Procedure B) and with concentration of eggs by sieving alone (Procedure C, Figure S2). As sugar gradient purification did not improve PCR results, Procedure C was chosen as the standard protocol and has been used for all subsequent analyses unless indicated differently.

### Identification of Specific Trichostrongylid Species by d-PCR

**Figure 2:**
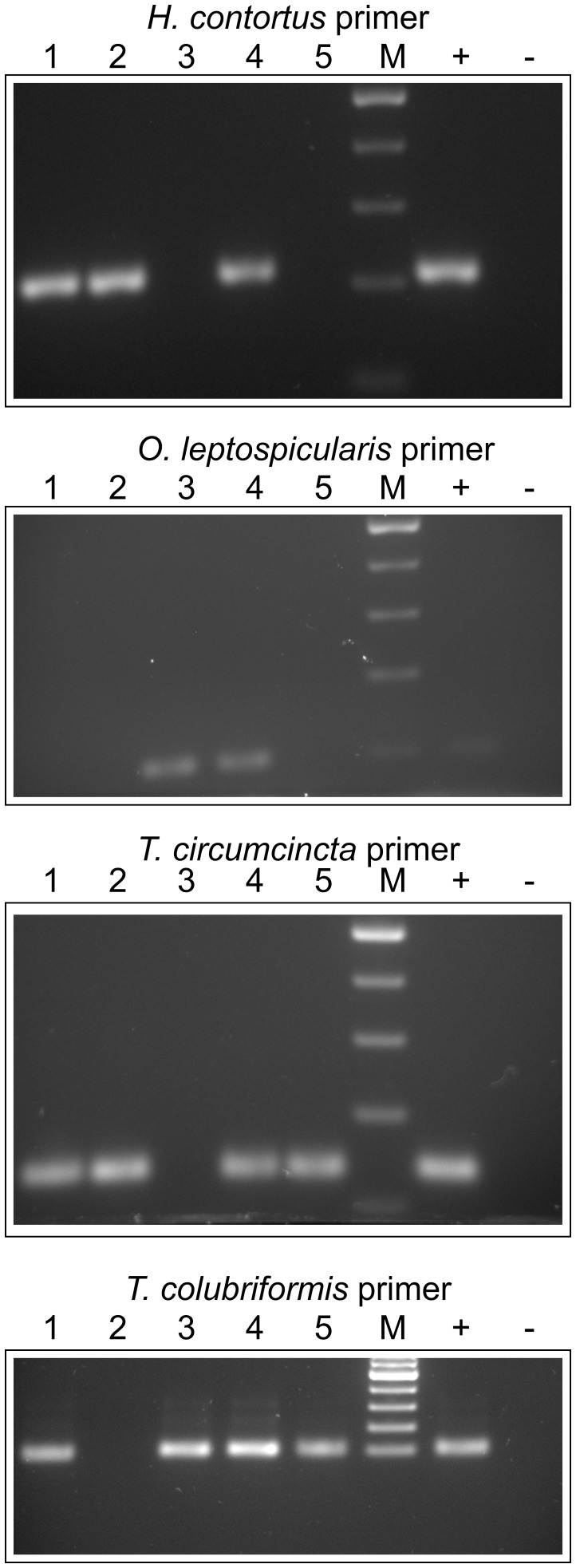
Qualitative identification of trichostrongylid nematodes of goats. Eggs were purified from five different animals (goats numbered 1 to 5 with epgs of 1241, 178, 307, 210, and 65, respectively) using the final protocol with sieving but without sucrose gradient. Lanes 1–5 present results for the individual goats. Primer pairs used are indicated above each gel. Positive controls (+) contained 1 ng plasmid DNA with the ITS-2 of the target species cloned in pCR4TOPO. Negative controls (−) contained only water. M, marker (100 bp ladder, Fermentas). PCRs were performed at least three times producing identical results and PCRs from extracted DNA (directly from eggs) also identified the same species. All PCR fragments were verified by sequencing.

Primer pairs specific for regions in the ITS-2 of *Haemonchus contortus*, *Teladorsagia circumcincta*, *O. leptospicularis*, *Trichostrongylus colubriformis*, *O. ostertagi* and *C. oncophora* were designed and first evaluated against ITS-2 plasmid DNA to establish conditions, where no cross reactivity could be observed. For this purpose, annealing temperature gradients were run for all primer pairs against the ITS-2 sequences of all above mentioned parasites. Figure S3 shows PCR reactions demonstrating absence of any cross-specificity for these species. Goat feces obtained from five different animals with an epg between 65 and 1241 (measured by FLOTAC with a sensitivity of 1 epg) were analyzed with these primer pairs revealing the presence of *H. contortus* (three out of five animals), *O. leptospicularis* (two out of five), *T. circumcincta* (three out of five), and *T. colubriformis* (three out of five) (Figure 2). At a second time point, feces of four animals were analyzed, two goats 14 days after treatment with moxidectin (Cydectin®) (without detectable epg) and two untreated goats (1734 and 128 epg, respectively). PCR analysis did not detect any of the four nematodes in the two moxidectin-treated animals (Figure S4). The goat with 128 epg feces was also negative by direct PCR for these four specific pathogens. Therefore, PCR with a primer pair specific for a partial fragment of the ITS-1 of trichostrongylids was performed for all four samples (data not shown). While both Cydectin®-treated animals remained negative by PCR, the goat with 128 epg feces was positive in the ITS-1 PCR.

### Proof of Principle for Feces from Other Host Species and Nematode Groups

**Figure 3:**
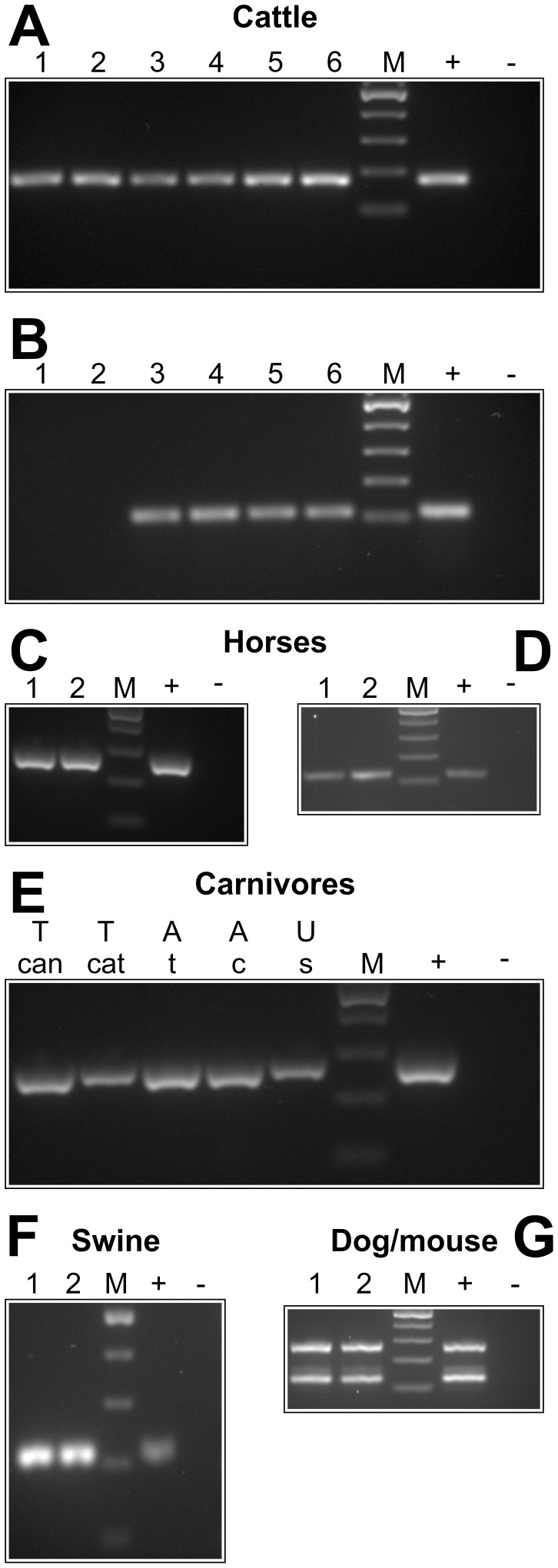
Compatibility of d-PCR with fecal samples from different animal species. Cattle samples were analyzed with primers for *C. oncophora* (A) and *O. ostertagi* (B). The 28S rDNA (C, E), the ITS-1 (D) and the ITS-2 (F) primer pair were used for feces of horses, carnivores and swine. A *Trichuris*-specific ITS-2 primer pair was used to detect *T. muris* and *T. vulpis* in murine and canine fecal samples (G). Lanes with different numbers represent individual animals. Positive controls (+) contained 1 ng plasmid DNA with an insert containing the corresponding target sequence. Only for *Trichuris*, genomic DNA isolated from *T. vulpis* eggs was used as positive control., negative controls were set up with water instead of template. M, marker (100 bp ladder, Fermentas); T can, *T. canis*, T cat, *T. cati*; A t, *A. tubaeformae*, A c, *A. caninum*, U s, *U. stenocephala*.

In order to evaluate whether the method works in principal also for other host species, nematode eggs from cattle, horse, swine, dog, cat and mouse feces were processed using the standard protocol. Calves were infected with three different *C. oncophora* isolates that are routinely passaged in our laboratory. While one of these isolates is pure, the other two were known to contain minor amounts (<10%) of *O. ostertagi* as revealed by larval culture. Cattle samples were analyzed by PCR with primers specific for *C. oncophora* (Figure 3A) or *O. ostertagi* (Figure 3B). While *C. oncophora* could be detected in all six calves, only samples from those four calves infected with the contaminated *C. oncophora* isolates were also positive for *O. ostertagi* DNA.

Fecal samples of two horses with an epg of about 25 (only strongylid egg type) were successfully subjected to PCR using both the 28S and the partial ITS-1 primer pair (Figure 3C, D). Direct sequencing of one 28S and one ITS-1 PCR product revealed 100% identity with *Cylicocylus insigne* 28S (accession number: AM039734) and 93% identity with *Cylicocylus nassatus* (accession number: Y08585). As shown in Figure 3E, detection of nematode DNA was also successfully performed with dog and cat feces containing *A. caninum*, *U. stenocephala*, *T. canis*, *A. tubaeformae* and *T. cati*, respectively.

Due to its high fat content swine fecal samples are often difficult to analyze using standard coproscopic methods. Nevertheless, strongylid type eggs could easily be quantified using FLOTAC and amplification with primers specific for the strongylid ITS-2 region was successful (Figure 3F). Sequencing identified *Oesophagostomum dentatum*. Pooled fecal samples from mice and dogs positive for *T. muris* and *T. vulpis* were used to address compatibility of the standard protocol with trichurid eggs. Although concentration of the eggs was possible without any modification, it was not possible to crack the *Trichuris* eggs by freeze boiling indicating a superior stability of *Trichuris* eggs compared to eggs of ascarids and strongylids. Mechanical disruption by heavily shaking in the presence of beads was required to release sufficient DNA to successfully perform PCR. After disruption, PCR with *Trichuris* specific primers for amplification of the ITS-2 region was achieved. However, a small non-specific band was co-amplified both in fecal extracts and with the positive control (genomic *T. vulpis* DNA) **(** Figure 3G).

### Evaluation of PCR Efficacies by Real-time PCR Directly from Fecal Samples

**Figure 4:**
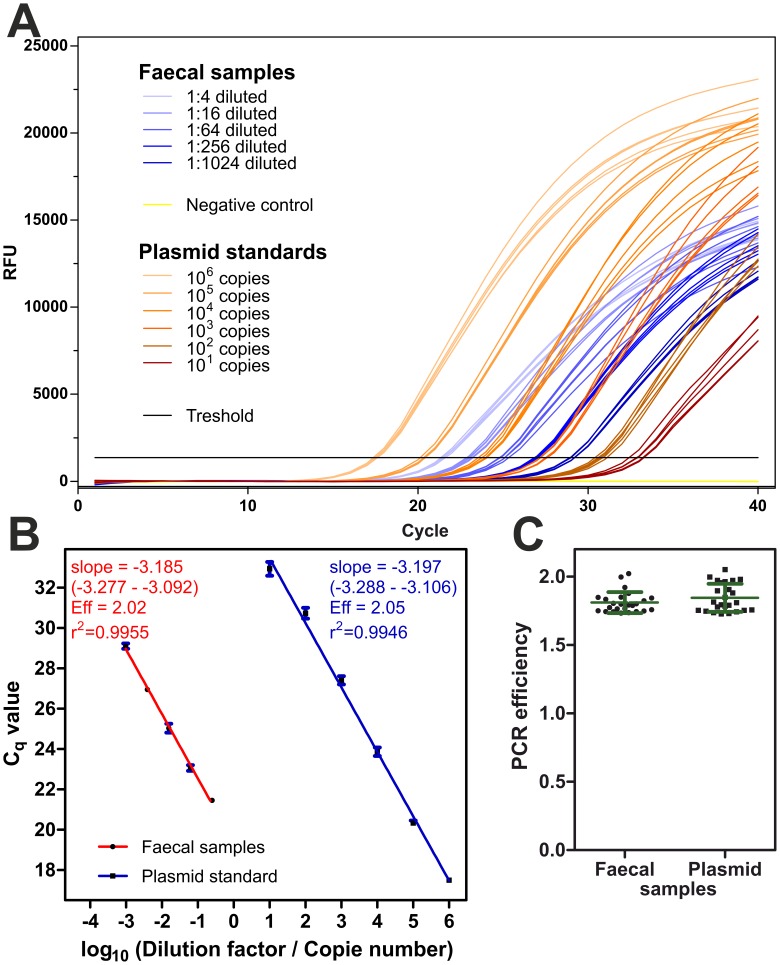
Determination of amplification efficacy by real-time PCR. Fourfold serial dilutions of fecal sample extract from a *T. cati* infected cat (1∶4, dilution factor 0.25 to 1∶1024, dilution factor 0.00098) and tenfold serial dilutions of plasmid DNA (10^6^ to 10^1^ copies) were used as template for real-time PCR in the presence of EvaGreen fluorescence dye. (A) Amplification plots showing signal accumulation measured in relative fluorescence units (RFU) with increasing cycle number. (b) Regression curves for fecal samples (red) and plasmid DNA (blue) were calculated with GraphPad Prism 5. Goodness of fit in terms of R^2^ and slopes (with 95% confidence intervals) are given and slopes were used to calculate PCR efficiencies (Eff). Both regressions curves are virtually parallel and no significant difference between slopes could be found. (c) Amplification efficacy was also calculated from the slopes of individual amplification plots with LinRegPCR. Individual efficacies for all fecal (dots) and all plasmid samples (squares) as well as means ± SD are presented. A Student’s t test was used to compare PCR efficiencies between both groups but no significant differences could be detected.

PCRs were analyzed for the presence of inhibitors by dilution of the template as suggested by the MIQE guidelines for quantitative real-time PCR [22]. Feces from a cat naturally infected with *T. cati* were used as an example to perform real-time PCRs. In parallel tenfold serial dilutions of plasmid DNA containing the 28S rDNA fragment of *T. cati* as insert and fourfold serial dilution of extracts from cat feces obtained using the standard protocol were processed. Since preliminary experiments revealed that undiluted fecal extracts showed variable C_q_ values that were not much lower than those of fourfold diluted samples, the final experiments were performed with extracts diluted at least 1∶4. RFUs were plotted vs. cycle number (Figure 4A). On the abscissa, either absolute copy number (plasmid samples) or dilution factors (fecal samples) were used. Background subtraction, threshold and C_q_ determination were performed in CFX manager 2.0. Detection of *T. cati* DNA in fecal samples was reliably achieved even when samples were diluted 1∶1024, corresponding to less than 0.003 eggs in the PCR reaction. Eggs freshly released from the uterus of females from the related parasite *A. suum* have been reported to contain on average 42.2 rDNA copies per egg, based on quantification using a primer pair targeting the ITS-1 region [23]. However, copy numbers increased rapidly during embryonation within the first 8 days to 8.8×10^3^. If the same copy number for *T. cati* as for *A. suum*, rDNAs in the genome and fully embryonated eggs are assumed, the PCR would be able to reliably detect 26.4 copies of the rDNA gene.

Semi-logarithmic regressions were calculated in GraphPad Prism 5 since this statistic tool allows comparison of different regression curves (Figure 4B). Dilution factors for fecal samples are shown on the same axis as absolute copy numbers for plasmid DNA. PCR efficacies were estimated on one hand by serial dilutions of template followed by calculation of a regression curve (Figure 4B). In cases of PCR inhibition by components in the crude fecal egg extracts, the efficacy calculated from the slope of the regression curve should be lower in fecal samples than with pure plasmid as template. Moreover, the correlation coefficient for the regression should be lower for fecal samples because increasing dilution should increase PCR efficacy. Unequal PCR efficacies between different target concentrations would cause deviation from linearity and thus decreased R^2^ values. There was no significant difference between slopes of regression curves (p = 0.89) with PCR efficacies very close to 100%. Coefficients of determination R^2^>0.99 indicate that there is no deviation from linearity. Thus, equal PCR efficacies can be assumed.

In parallel, LinRegPCR 11.0 was used to calculate PCR efficacies from individual reactions. C_q_ values for individual amplification curves were slightly lower than those obtained from regression analysis of dilution series (Figure 4C). Comparison between all fecal samples and all plasmid samples using a Student’s t test revealed no significant differences (p = 0.38). Therefore, for extracts diluted at least 1∶4, no PCR inhibition was detected.

### High Resolution Melt Analysis to Differentiate PCR Products

**Figure 5:**
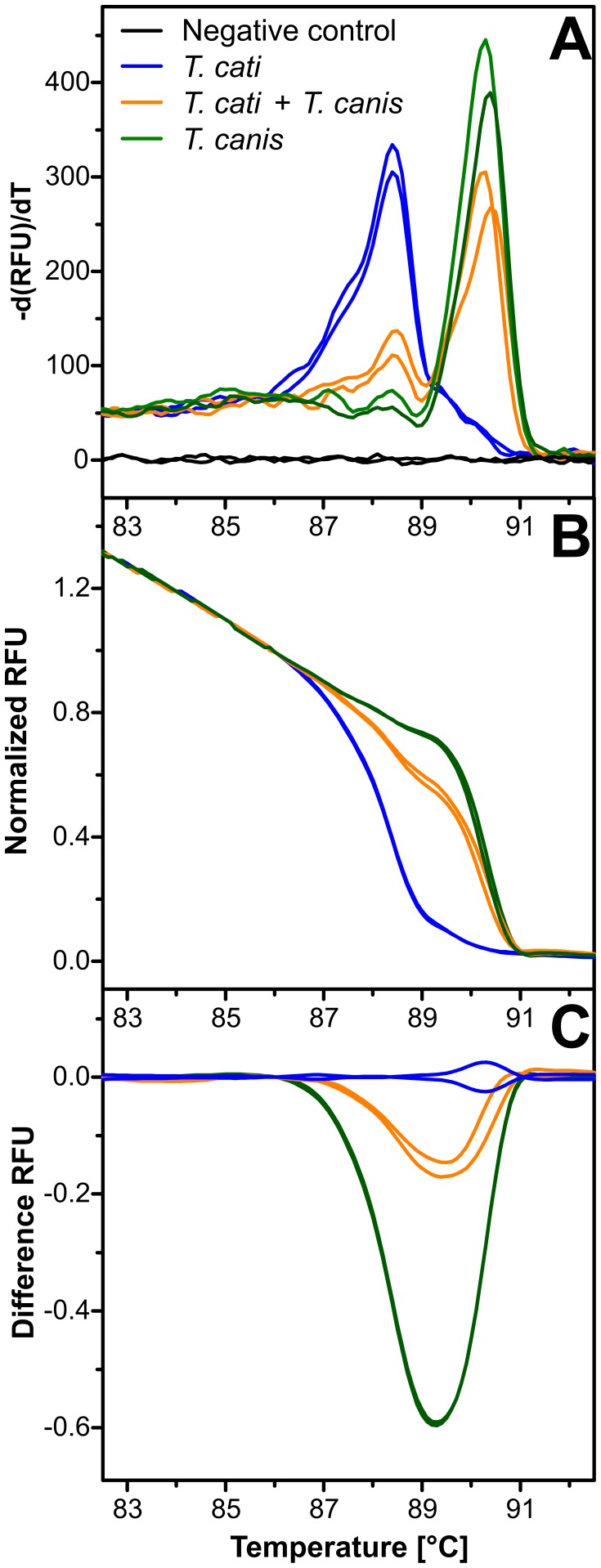
Parasite species identification by melting curve analysis. Fecal extracts from samples containing *T. canis*, *T. cati* or a mixture of both species were used as template for real-time PCR in the presence of EvaGreen followed by a high resolution melting curve analysis. Melting peaks obtained from maxima in the plot of the first deviation of the fluorescence intensity d(RFU)/dT were 88.4–88.5°C for *T. cati* and 90.3–90.4°C for *T. canis* (a). Amplicons from the mixed samples showed both peaks. Melting curves were normalized (b) and a difference plot (c) was calculated by subtracting the mean of both *T. canis* samples from all individual curves. Results for one of three representative experiments are shown.

In order to differentiate the morphologically highly similar eggs of *T. canis* and *T. cati*, a high resolution melt (HRM) analysis was established using feces from dogs and cats infected with *T. canis* or *T. cati*, respectively. The potential of the 28S primer pair was evaluated for HRM-based species identification. The first derivative of melting curves (d(RFU)/dT) for amplicons obtained from *T. canis*, *T. cati* and double-positive fecal extracts are shown in Figure 5A. Melting peaks for both species differ clearly and both peaks can be observed in the double positive mixture. After normalization of melting curve data (Figure 5B) and in particular in a difference plot with mean *T. canis* data subtracted from all individual curves (Figure 5C) species can be unequivocally identified with this method. The clustering tool implemented in the Precision Melt Analysis Software identified three different clusters corresponding to the three types of samples in the melting curves shown in Figure 5 and confidence levels for these assignments were between 98.8% and 99.8%.

### Sensitivity and Possible Correlation between epgs and C_q_ Values

**Figure 6:**
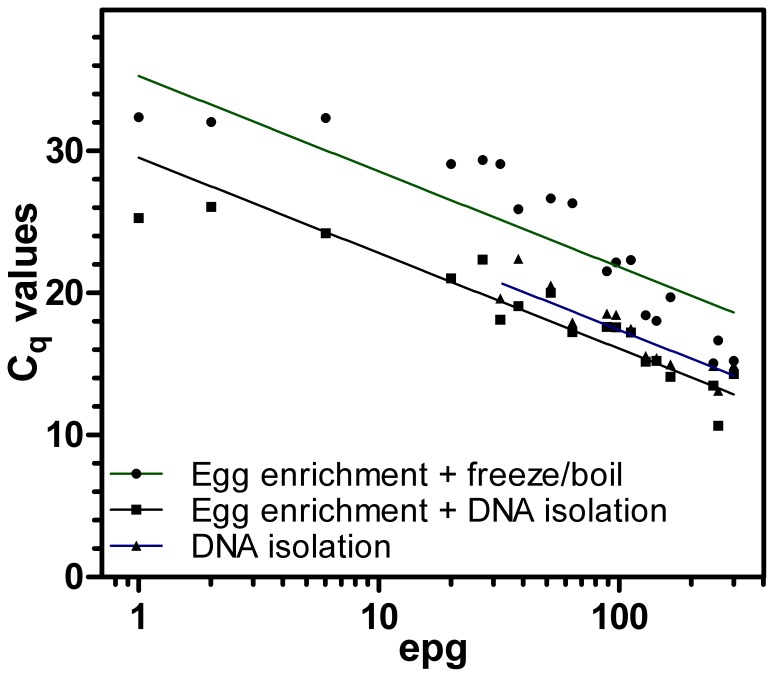
Comparison of different template preparation methods. Fecal samples negative for nematode eggs were spiked with *C. oncophora* eggs aiming to obtain epgs of 250, 150, 100, 50, 25 and 5. Every sample was split in three parts and analyzed either by direct DNA isolation from 500 mg feces using a commercial kit or by concentration of eggs from 10 g feces including determination of actual epgs by FLOTAC. Eggs were either obtained by flotation followed by DNA extraction or by flotation and sieving followed by freeze-boiling (d-PCR). C_q_ values were plotted vs. actual epgs as determined by FLOTAC and semi-logarithmic regression curves were fitted in GraphPad Prism software.

Fecal samples spiked with *C. oncophora* eggs to achieve epgs between 5 and 250 were processed using Procedures C (d-PCR after egg concentration by flotation and sieving followed by freeze boiling as used throughout this study), D (egg concentration by flotation followed by DNA isolation) and E (direct DNA purification from 0.5 g of fecal matter) as shown in Figure 1. Equal aliquots were then subjected to real-time PCR using the 28S rDNA primer pair. Actual epgs as determined by FLOTAC varied between 1 and 301. All samples were positive using d-PCR (Procedure C) and egg concentration followed by DNA isolation (Procedure D). In contrast, 32 eggs per gram was the sample with the lowest epg that was positive with direct DNA purification (Procedure E) while all five samples with epgs between 1 and 27 remained negative. Although C_q_ values were in general lower for egg concentration followed by DNA extraction (Procedure D) than for d-PCR (Procedure C), both methods were reliably able to detect nematode DNA with epgs below 10. C_q_ values were then plotted vs. actual epgs and semi-logarithmic regressions were calculated (Figure 6). As can be expected, variability was highest for samples which were spiked to obtain a very low epg of 5. Therefore regression curve analysis was performed twice, once with all samples and once excluding these samples. Using only samples with epgs of at least 20, coefficients of determination (R^2^) were 0.83 for both Procedures D and E and 0.94 for Procedure C. If samples with very low epgs (between 1 and 6) were also included, R^2^ values of 0.88, 0.81 and 0.83 were obtained for Procedure C, D and E, respectively. Thus there is a strong correlation between egg counts and C_q_ values in real-time PCR and even in samples with epgs below 10, both procedures (C and D) reliably detected nematode DNA.

### Diagnosis of Human Samples

Finally the method was adapted for analysis of human stool samples to allow the use of the method for tropical human medicine. Since human samples positive for gastrointestinal nematode eggs are rarely found in industrialized countries such as Germany, we had to rely on conserved samples collected in tropical regions.

For evaluation of compatibility with human samples only very small volumes (100 µl) were initially available. Moreover, these samples had already been subjected to three cycles of freezing and thawing before arrival in our laboratory. The flotation method was downscaled, however, no nematode eggs were found in the enriched material. Nevertheless, d-PCR was tried using the ITS-2 and 28S primer pairs. Weak amplification of fragments with the expected size was possible, however, bands were faint and duplicate PCR reactions rarely gave identical results. Thus results remained largely non-reproducible (data not shown).

In order to evaluate the d-PCR method for analysis of human samples, import of samples from tropical areas with high prevalence of gastrointestinal nematodes was initiated. Different conservation methods were then evaluated for compatibility with both FLOTAC and d-PCR using *C. oncophora* eggs. Neither freezing (once) nor fixation in formaldehyde, ethanol or potassium dichromate allowed both detection of intact eggs in FLOTAC chambers and amplification of target DNA by PCR (data not shown). In contrast, mixing with 5–6 volumes 1% Lugol’s iodine solution conserved the samples without interfering with amplification.

**Figure 7:**
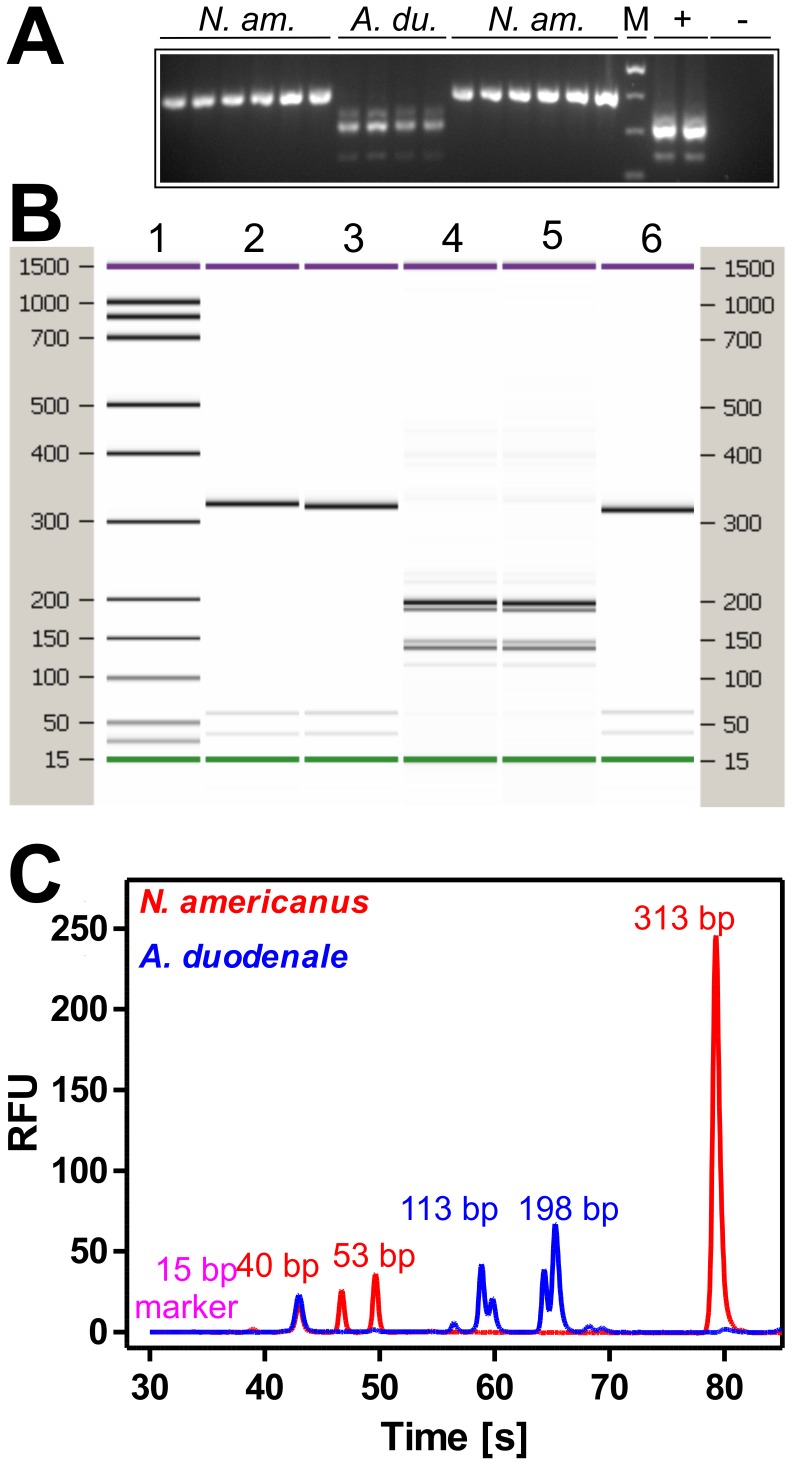
Species determination for human hookworm samples. (A) Eight human and one canine (*A. caninum*) sample (+) together with hookworm negative fecal extract (−) were analyzed in duplicate using the ITS-2 primer pair. *N. am.*, *Necator americanus*; *A. du.*, *A. duodenale*; M, marker (100 bp ladder, Fermentas). (B, C) Representative PCR products were purified and 150 ng were digested with *Rsa*I and separated using the DNA1000 LabChip® which has a sizing range from 25–1000 bp. Theoretical fragment sizes for *N. americanus* are 313, 53, 40 and 11 bp and 159, 139, 35, 28, 28, 16 and 12 bp. In silico digestion of *A. duodenale* or *A. caninum* fragments leads to identical fragment sizes of 198 and 113 bp. The gel view is shown in (B) for five samples and the electropherogram in (C) for two samples.

Therefore, human stool samples collected for diagnostic purposes in Tanzania were fixed with Lugol’s iodine and send to Berlin. Transport of the samples without refrigeration took more than three weeks. Nevertheless, analysis of several hookworm positive samples (epg between 60 and 400) with strongylid ITS-2 specific primers resulted in amplification of a DNA fragment slightly larger than 400 bp in most of the samples already suggesting the presence of *Necator americanus* (Figure 7A). In a few samples a fragment slightly larger than 300 bp as expected for *Ancylostoma* spp. was detected. In one sample both fragments were amplified simultaneously (Figure 7A). Additionally, a weak amplification product slightly smaller than the predominant ITS-2 fragment was detected in all samples positive for *A. duodenale* as well as in the positive control containing *A. caninum*. Presence of a minor fraction of shorter ITS regions due to intragenomic variability has previously been reported in *A. duodenale* [24]. The use of hookworm-negative samples did not result in any amplification. In order to confirm species identification, RFLP analysis was performed for selected human samples. Since many of the expected fragment sizes are well below 100 bp in size, the Bioanalyzer 2100 was used to separate the restriction products (Figure 7B and C) although 2.5% agarose gels are also able to provide unequivocal results. In all human samples presence of *N. americanus* or *A. duodenale* was confirmed. Three selected PCR products were further confirmed by sequencing. Two were 100% identical to *N. americanus* sequences deposited in GenBank® (e.g. accession no. JF960388) and one to *A. duodenale* (e.g. EU344797) and therefore confirmed PCR and RFLP results. The high resolution melt technique was also applied to human hookworm samples (Figure S5). With one exception, all samples were correctly clustered by the Precision Mel Analysis software with 98.3–99.8% confidence. Visual inspection of the only *N. americanus* positive sample that did not cluster with the other PCRs – including its own technical replicate, revealed a small shift in the melt curve towards higher temperatures. Nevertheless, the shape of the melt curve was clearly of the *N. americanus* type with a single melting domain and not of the *A. duodenale* type with three melting domains due to the three different ITS-2 amplicons. It was not possible to unequivocally distinguish *A. duodenale* and *A. caninum* with this method.

## Discussion

The direct PCR method developed in this project has the potential to allow highly sensitive molecular diagnosis of gastrointestinal nematodes with importance for veterinary and human tropical medicine at decreased costs. Compared with other already published methods this protocol shows similar sensitivity and reproducibility without increased handling efforts but the benefit of no DNA isolation. Since no DNA isolation has to be incorporated, no additional costs of e.g. 3.35 € per reaction for the NucleoSpin® Soil DNA extraction kit (Macherey-Nagel) have to be calculated. The protocol described here is highly versatile regarding the source of fecal matter being compatible with samples from herbivore (ruminants, horses), omnivore (swine, humans) and carnivore (dogs, cats) hosts. One important step in the present protocol is the direct use of the fecal suspension obtained after sieving, flotation and washing. Despite the washing and sieving steps, which predominantly serve to concentrate the eggs in a small volume, this suspension is far from being purified or “clean” and therefore the use of an inhibitor-resistant polymerase is crucial. Phusion and Phusion II have been claimed by the manufacturer to be tolerant to many PCR inhibitors and allow direct amplification from blood and tissues. Indeed direct PCR from blood, semen, saliva and hair roots for forensic purposes was recently reported by Verheij et al. [25]. To our knowledge, however, this is the first study showing that PCR efficacies close to the theoretical maximum can be achieved from inhibitor-rich fecal samples without any DNA purification step. Our results show that Phusion II is as well superior to Taq DNA polymerase when dealing with inhibitors found in feces. Schnieder et al. [10] have reported amplification directly from individual nematode eggs using Tfl DNA polymerase but that could not be reproduced by Harmon et al. [11] using Taq DNA polymerase. One explanation for this observed discrepancy might be that these polymerases also differ in their susceptibility to PCR inhibitors.

Another important advantage is that the sample volume can be increased considerably up to at least 100 g without any significant additional costs and efforts. Most commercial DNA isolation kits can only be used with small fecal samples (<1 g) and are usually only evaluated for extraction of host, viral and bacterial DNA. Such DNA purification protocols are limited in their sensitivity by the small amount of fecal input material which is even more relevant since nematode eggs are not evenly distributed in feces. Moreover, recent results have shown for coccidian oocysts, that kits from different suppliers differ significantly in their efficacy to extract parasite DNA from fecal samples [19]. Since nematode eggs and oocysts of coccidia exhibit similar tenacity, it should at least be taken into consideration that different DNA isolation kits might also differ substantially in their efficacy to extract DNA from nematode eggs in fecal samples. Different nematode egg types might also be more or less susceptible to lysis by repeated boil-freeze cycles. While this lysis method worked well for strongyle and ascarid eggs, it was insufficient for *Trichuris* spp. eggs. Nevertheless, in order to use this protocol for quantification of species present, careful evaluation of the method for different nematode species/genus/families will be required.

For diagnosis of *Trichuris* eggs molecular techniques are usually not required, since due to their characteristic morphology the eggs can easily be distinguished from those of other nematode species. Additionally whipworms are usually very host specific and thus only one *Trichuris* species occurs per host species (except ruminants). However, morphological differentiation is not possible for e.g. the many ruminant or horse strongylid nematode species eggs and at least extremely difficult for e.g. most carnivore ascarid eggs. Accordingly the design of species specific primers is required to identify the individual species present in the sample. This can either be achieved by using conventional PCR and gel electrophoresis or by real-time PCR with species specific probes as described for nematodes of small ruminants [14], [15]. As an alternative, primers directed against highly conserved sequences can be used followed by post PCR analyses for species identification. For mono-species infections, Sanger sequencing is sufficient. However, infections of horses with small strongyles are always mixed infections with multiple species and also in ruminants nematode infections are predominantly caused by multiple species. For such multi-species mixed infections sequencing can of course only detect the predominant species and when several species are present in approximately equal amounts, even unreadable chromatograms might be obtained. Combination of d-PCR with primers flanking the intergenic or internal transcribed spacers followed by established reverse-line-blot methods [26], [27] and by quantitative image analysis should allow the rapid semi-quantitative characterization of complex population structures of strongyles in horses at a molecular level. Additional options, in particular if the number of suspected species is relatively low, are RFLP and HRM analyses as demonstrated herein for discrimination of *Toxocara* and hookworm species. The main advantages of RFLP are that it is simple, cheap and does not require any sophisticated equipment. HRM allows rapid differentiation of highly similar PCR products – sometimes differing only in a single nucleotide polymorphism – without additional hands-on time after PCR – and has been successfully used for diagnosis of pathogen species [28] including parasites [29], [30] in diagnostic samples. Even genotyping of parasites to analyze population structure or presence of resistance markers in parasites has been reported [31], [32], [33].Whereas HRM has often been successfully applied to specifically identify closely related pathogens in diagnostic samples, it has clear disadvantages when mixed-infections with more than two pathogens have to be taken into account since this might result in very complex melting curves which are difficult to interpret. However, for host species which are not frequently infected with multiple species of gastrointestinal nematodes, HRM might offer a fast and very reliable method to identify the parasites.

For two nematodes with significant relevance for human health we have developed protocols that allow their discrimination by d-PCR. Human toxocarosis is a very widespread neglected poverty-associated disease both in regions with tropical and moderate climate [34], [35]. In humans, ingestion of *Toxocara* eggs shed by various carnivore hosts can cause a larva migrans visceralis syndrome. Identification of the relevant host species (*e.g.* dogs, cats, foxes) that significantly contribute to contamination of the environment is an important prerequisite to conduct successful intervention measures. Since the egg morphology of *Toxocara* species is relatively similar, it is easily possible to misidentify the most important species, *T. canis* and *T. cati* by classical coproscopical diagnosis. In fecal samples of dogs, presumably due to coprophagy, *T. cati* eggs have been reported to be present in nearly one third of all *Toxocara* positive dogs in Switzerland [36]. Thus, *T. cati* might contribute to a significant percentage of those non-juvenile dogs found to be frequently positive for *Toxocara* spp. [36]. From an epidemiological point of view, the distinction between *T. cati* and *T. canis* in carnivore feces is therefore very important and the HRM method described here provides rapid and sensitive tool.

For hookworms a new HRM analysis was developed that is able to discriminate *Ancylostoma* and *Necator*, and a RFLP method for discrimination to the species level was used. Discrimination of *A. duodenale* and *N. americanus* is relevant from an epidemiological point of view. Although only a few datasets have been published yet comparing different hookworm species regarding drug susceptibility, there is obvious evidence that *N. americanus* and *Ancylostoma* spp. differ strongly in their susceptibility to drugs of various classes. *N. americanus* was three times more susceptible to thiabendazole than *Ancylostoma ceylanicum* in an egg hatch *in vitro* assay [37] and 50 times more susceptible to ivermectin in feeding and motility assays [38]. In contrast, EC_50_ values for responses to pyrantel where nearly 100 times higher for *A. ceylanicum* than for *N. americanus* in a motility assay [37]. *In vivo*, *N. americanus* has been reported to be 300 times less susceptible to ivermectin than *A. ceylanicum* whereas no differences in response to pyrantel were observed [39]. Thus, identification of the endemic hookworm species and particularly of the species remaining after mass drug treatments is of high interest for further treatment decisions.

### Conclusions

The results presented here show that PCR products produced by d-PCR are amenable to analysis by RFLP, Sanger sequencing and real-time PCR with fluorescent dyes binding double-stranded DNA followed by HRM analysis to rapidly identify particular pathogens. There is no reason to believe that it should not as well be possible to use them for hybridization-based methods such as reverse-line-blot or probe-based real-time PCR applications. This will further expand the power of the method. Due to the diverse host species that were used in the present study, including herbivores, carnivores and omnivores with quite different composition of feces, it can be assumed that at least most mammalian fecal samples can be analyzed with the method described here in detail. The demonstration that d-PCR is suitable for analysis of human fecal samples extends the broad applicability of the method to tropical medicine. Although the most important human gastrointestinal nematodes can be readily distinguished microscopically and therefore one might argue that molecular diagnostic tools are not required in this field, we believe that molecular tools can significantly improve our epidemiological insights and improve both surveillance and treatment decisions. For example molecular tools are required to discriminate zoonotic *A. suum* from anthroponotic *Ascaris lumbricoides* in humans [40] or to monitor the level of β-tubulin alleles conferring drug resistance in worm populations under high selection due to mass treatment programs with benzimidazoles [41]. Detection or even quantification of alleles conferring resistance to anthelmintics directly from fecal samples pre and post treatment is an obvious application of the d-PCR with high relevance to veterinary and tropical human medicine.

## Supporting Information

Figure S1 Microscopic examination of purified strongylid eggs from goat feces before and after lysis by boiling and freezing. Trichostrongylid type eggs from a sheep sample are shown in (A) which could *e.g.* be *Haemonchus*, *Ostertagia*, *Teladorsagia*, or *Trichostrongylus*. A typical egg suspension as obtained after three freeze/boil cycles is shown in an overview in (B) demonstrating the high amount of fecal debris which is still in the samples. (C) and (D) show boiled nematode eggs at higher magnifications. In (D) several empty eggs are visible and the cell mass from a single egg has been released from the shell.
(PDF)

Figure S2 Comparison of different DNA polymerases for direct fecal PCR. Eggs were purified from goat feces (one animal with approximately 500 epg, two replicates) by either purification over a sucrose step gradient and sieving or by sieving alone and re-suspension in 50 µl H_2_O followed by lysis using three freeze-boil cycles. Aliquots of 2 µl were subjected to PCR using either Maxima Hot Start Taq DNA polymerase (T) or Phusion DNA polymerase (P) and the primers Nematode-28Sfor and Nematode-28Srev. Positive controls (+) contained 1 ng plasmid DNA with the same amplicon from *T. colubriformis* in pCR4TOPO and negative controls (−) contained H_2_O as template. M, marker (100 bp ladder, Fermentas).
(PDF)

Figure S3 Evaluation of potential cross reactivity of species specific primer pairs against trichostrongylid parasites of small ruminants. The primer pairs used are indicated above the individual gels. All primer pairs were tested using plasmid DNA containing the ITS-2 sequence of *H. contortus* (Hc), *T. circumcincta* (Tci), *O. leptospicularis* (Ol) and *T. colubriformis* (Tco). For this purpose, annealing temperature gradients were run for all primer pairs against the ITS-2 sequences of all above mentioned parasites. At optimized PCR conditions as shown here, absence of any cross-specificity for these species could be shown. M, marker (100 bp ladder, Fermentas).
(PDF)

Figure S4 Reliable identification of animals without gastrointestinal nematodes. Eggs were purified from four different animals (goats numbered 1 to 4 with epgs of 1728, 0, 0, and 128. using the final protocol with sieving but without sucrose gradient. Both goats without eggs had been treated with the recommended dose of moxidectin (Cydectin®) 14 days before sampling of faeces. Primer pairs used are indicated above each gel. Positive controls (+) contained 1 ng plasmid DNA with the ITS-2 of the target species cloned in pCR4TOPO. Negative controls contained only water. M, marker (100 bp ladder, Fermentas).
(PDF)

Figure S5 Discrimination of human hookworm species using high-resolution melt PCR. The same samples as shown in Figure 5 were amplified in the presence of EvaGreen. High resolution melt curves were obtained at the end of the run. Raw melt curves (A), the first derivative of the melt curve (B), the normalized melt curves (C) and a difference plot (D) are shown. One *N. americanus* sample (only one of the technical duplicates, plotted in black) was not assigned into the same cluster as the other samples/replicates by the Precision Melt Analysis software.
(PDF)

Table S1 Primer pairs used for d- PCR.
(PDF)
