# Supplementary figures and images for: Discrimination of Gastrointestinal Nematode Eggs from Crude Fecal Egg Preparations by Inhibitor-Resistant Conventional and Real-Time PCR
Source: PLoS One. 2013 Apr 19;8(4):e61285. doi: 10.1371/journal.pone.0061285 (PMC3631180; doi:10.1371/journal.pone.0061285)

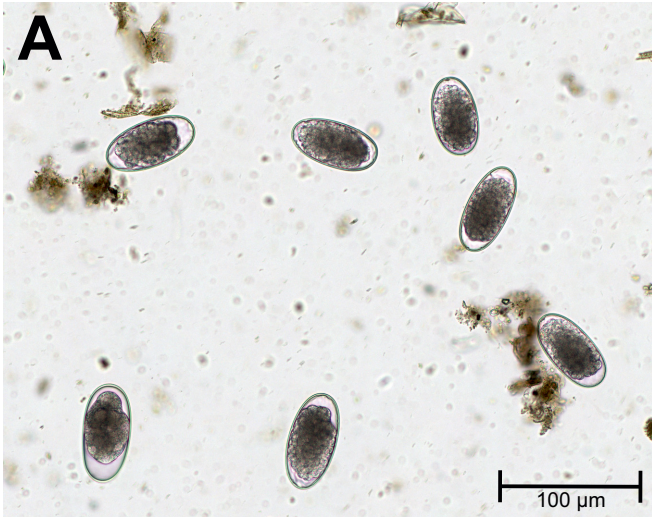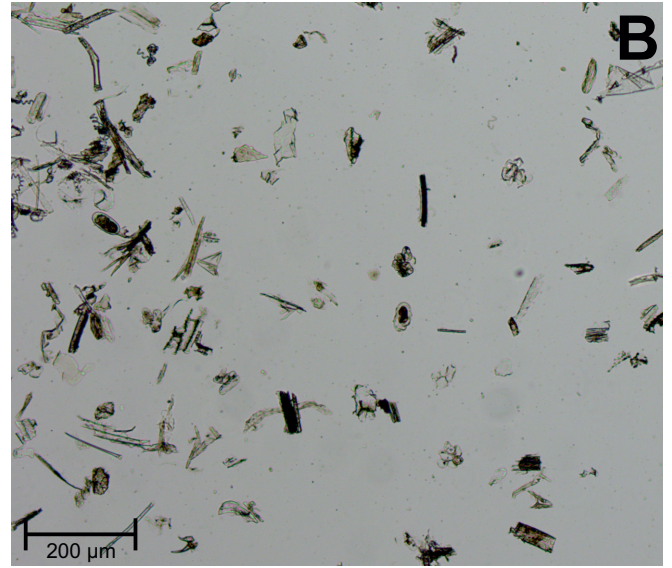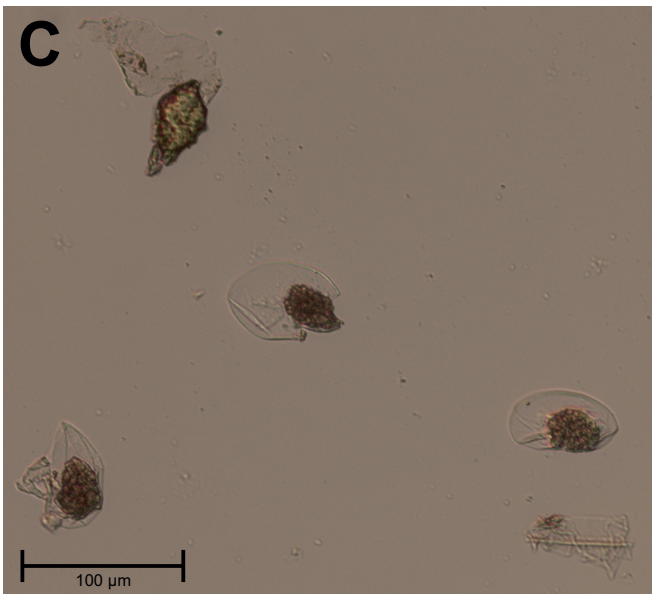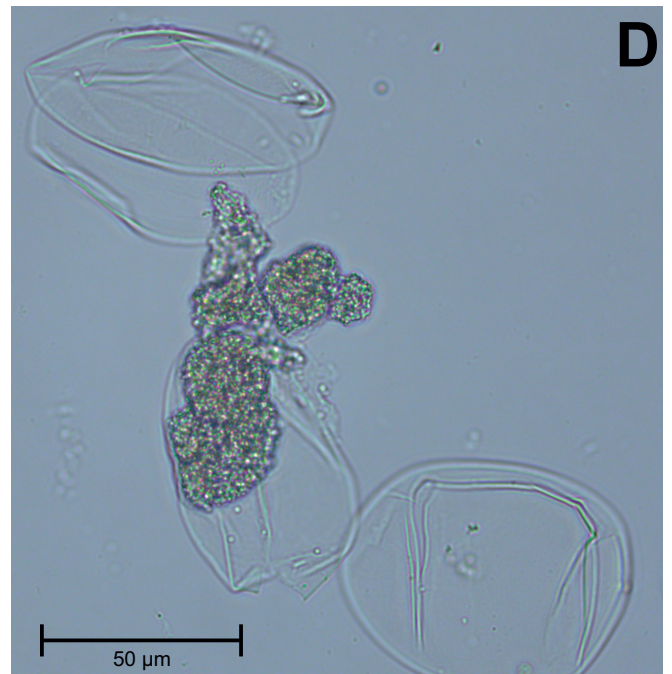

Supplement: Figure S1 — Microscopic examination of purified strongylid eggs from goat feces before and after lysis by boiling and freezing. Trichostrongylid type eggs from a sheep sample are shown in (A) which could e.g. be Haemonchus, Ostertagia, Teladorsagia, or Trichostrongylus. A typical egg suspension as obtained after three freeze/boil cycles is shown in an overview in (B) demonstrating the high amount of fecal debris which is still in the samples. (C) and (D) show boiled nematode eggs at higher magnifications. In (D) several empty eggs are visible and the cell mass from a single egg has been released from the shell. (PDF) [file pone.0061285.s001.pdf]

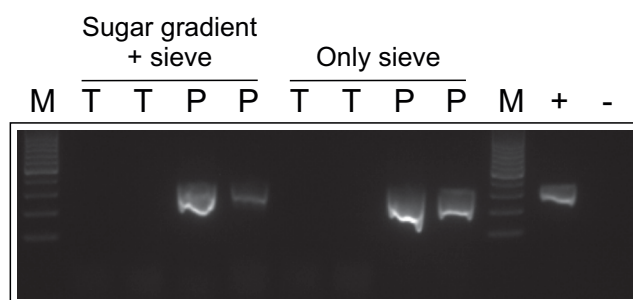

Supplement: Figure S2 — Comparison of different DNA polymerases for direct fecal PCR. Eggs were purified from goat feces (one animal with approximately 500 epg, two replicates) by either purification over a sucrose step gradient and sieving or by sieving alone and re-suspension in 50 µl H2O followed by lysis using three freeze-boil cycles. Aliquots of 2 µl were subjected to PCR using either Maxima Hot Start Taq DNA polymerase (T) or Phusion DNA polymerase (P) and the primers Nematode-28Sfor and Nematode-28Srev. Positive controls (+) contained 1 ng plasmid DNA with the same amplicon from T. colubriformis in pCR4TOPO and negative controls (−) contained H2O as template. M, marker (100 bp ladder, Fermentas). (PDF) [file pone.0061285.s002.pdf]

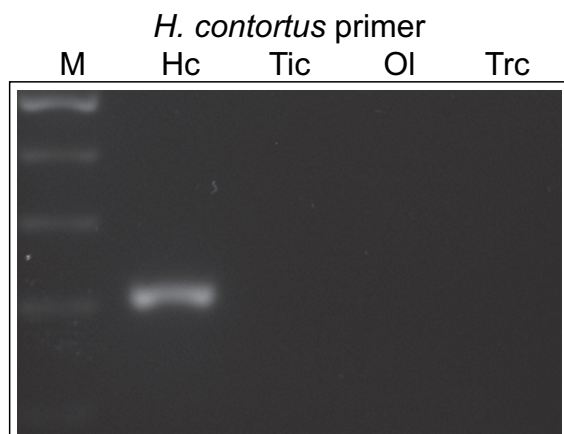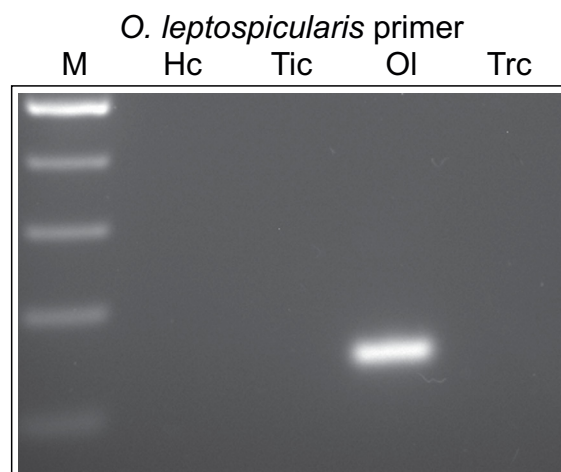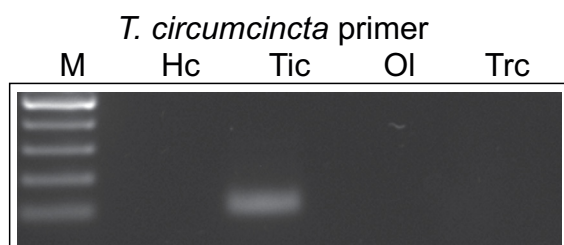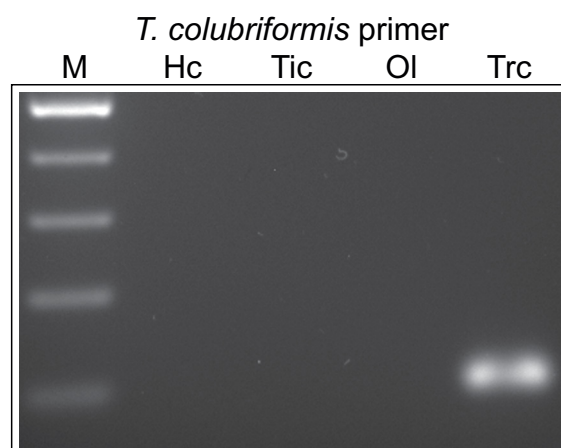

Supplement: Figure S3 — Evaluation of potential cross reactivity of species specific primer pairs against trichostrongylid parasites of small ruminants. The primer pairs used are indicated above the individual gels. All primer pairs were tested using plasmid DNA containing the ITS-2 sequence of H. contortus (Hc), T. circumcincta (Tci), O. leptospicularis (Ol) and T. colubriformis (Tco). For this purpose, annealing temperature gradients were run for all primer pairs against the ITS-2 sequences of all above mentioned parasites. At optimized PCR conditions as shown here, absence of any cross-specificity for these species could be shown. M, marker (100 bp ladder, Fermentas). (PDF) [file pone.0061285.s003.pdf]

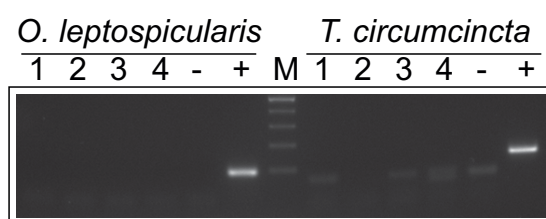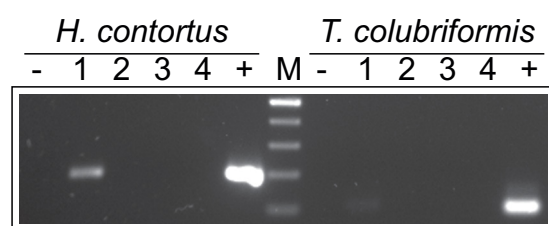

Supplement: Figure S4 — Reliable identification of animals without gastrointestinal nematodes. Eggs were purified from four different animals (goats numbered 1 to 4 with epgs of 1728, 0, 0, and 128. using the final protocol with sieving but without sucrose gradient. Both goats without eggs had been treated with the recommended dose of moxidectin (Cydectin®) 14 days before sampling of faeces. Primer pairs used are indicated above each gel. Positive controls (+) contained 1 ng plasmid DNA with the ITS-2 of the target species cloned in pCR4TOPO. Negative controls contained only water. M, marker (100 bp ladder, Fermentas). (PDF) [file pone.0061285.s004.pdf]

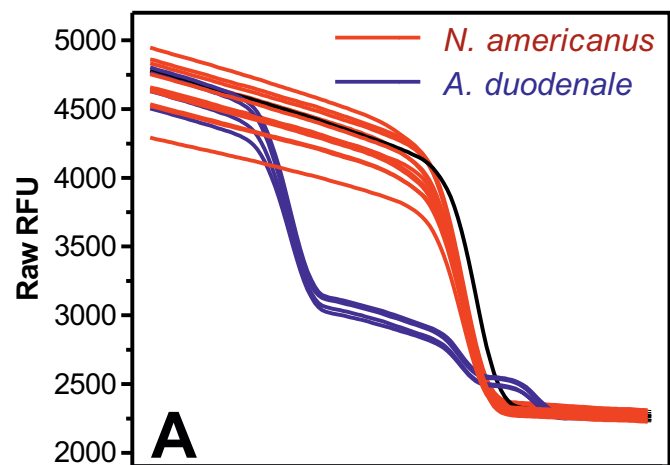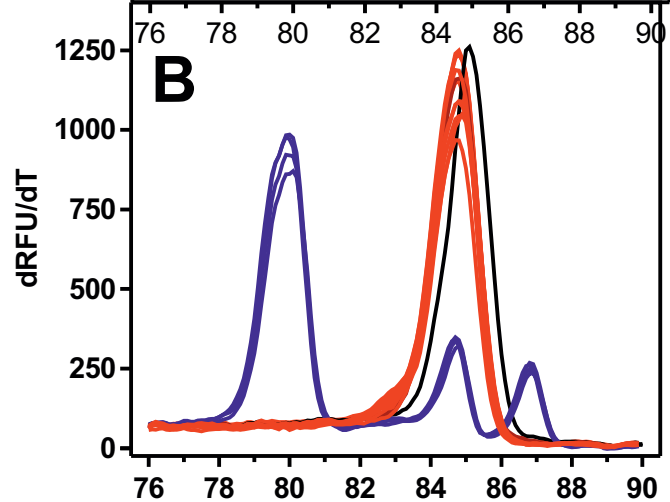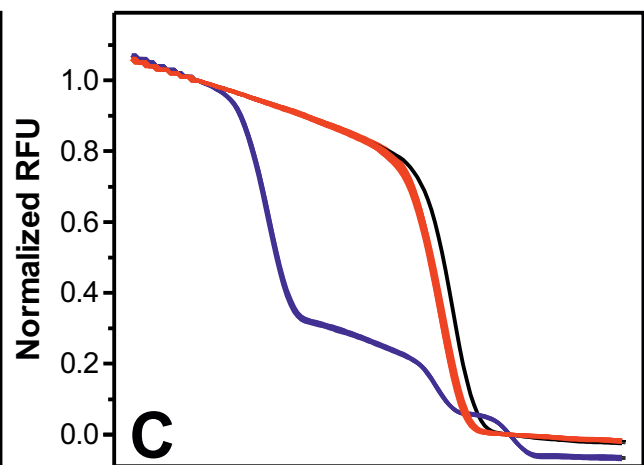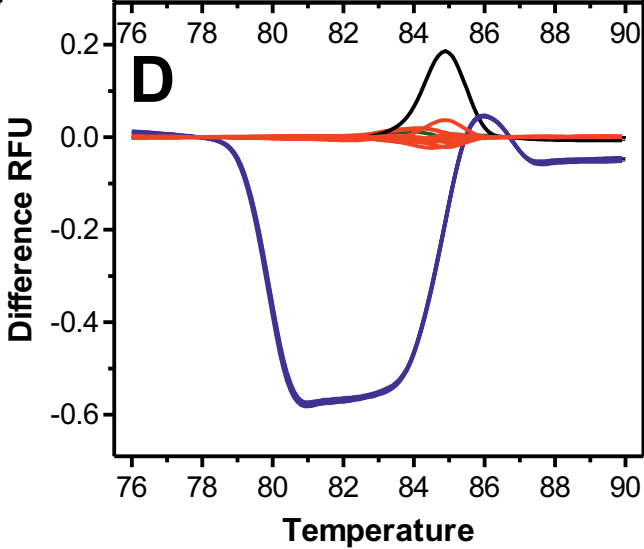

Supplement: Figure S5 — Discrimination of human hookworm species using high-resolution melt PCR. The same samples as shown in Figure 5 were amplified in the presence of EvaGreen. High resolution melt curves were obtained at the end of the run. Raw melt curves (A), the first derivative of the melt curve (B), the normalized melt curves (C) and a difference plot (D) are shown. One N. americanus sample (only one of the technical duplicates, plotted in black) was not assigned into the same cluster as the other samples/replicates by the Precision Melt Analysis software. (PDF) [file pone.0061285.s005.pdf]
