# Supplementary material for: Discrimination of Gastrointestinal Nematode Eggs from Crude Fecal Egg Preparations by Inhibitor-Resistant Conventional and Real-Time PCR
Source: PLoS One. 2013 Apr 19;8(4):e61285. doi: 10.1371/journal.pone.0061285 (PMC3631180; doi:10.1371/journal.pone.0061285)
Supplement: Table S1 — Primer pairs used for d- PCR. (PDF) [file pone.0061285.s006.pdf]

Table S1. Primer pairs used for d- PCR.

Name	Target	Sequence	Ta [°C][1]	Length [bp][2]
Hc-SH-for2<br>Hc-SH-rev	*H. contortus* ITS-2	CCATATACTACAATGTGGCTAATTTC<br>TACAAATGATAAAAGAACATCGTCGC	62	226
Ol-SH-For<br>Ol-SH-Rev	*O. leptospicularis* ITS-2	TTCCCATTTCAGTTCAAGAAAACAC<br>TTCATTGAGTACATTCAAATAGTGATAATATATA	60	106
Telc-SH-For1<br>Telc-SH-Rev1	*T. circumcincta* ITS-2	AACATATGCAACATGACGTACGACGG<br>ATGATACATTGAACATATATTACCATACATGTCT	53	147
Tricho-2-Multi-83F<br>Tricho-2-Multi-187R	*T. colubriformis* ITS-2	CTTACGTCTGGTTCAGGGTTG<br>ACTGAAATGGGAATCATCACAATATTT	53	106
Coop-SH-For2<br>Coop-SH-Rev2	*C. oncophora* ITS-2	ATGGCATTTGTCTACATCTGTTT<br>AAATGATAACGAATACTACTATCTCCA	62	192
Ost.ost-SH-For<br>Ost.ost-SH-Rev	*O. ostertagi* ITS-2	TAACATTGTTAACGTTACTGAATGATACTG<br>ATATAAATGATACATCGAATATACAATAC	50	124
Trichuris-ITS2-For<br>Trichuris-ITS2-Rev	Complete ITS-2	GCGGCAGTGTGGATCTGGCTG<br>GTGATCCGCCGTTCGGAATG	72	Species dependent
ITS1-part-For<br>ITS1-part-Rev	Partial strongylid ITS-1	TAGGTGAACCTGCAGATGGATCATCGTCGAAAC<br>AGAGCTYYAACGGGGGTGATACTCCCAAC	55	≈130
Nematode-28sfor<br>Nematode-28srev	Nematode 28S rDNA	GGCGAGTGAACGGGGAGAAGCCCAGCGCTGAA<br>TTTCCTTCACAGTACTTGTTTGCTATCGAATT	55	286
NC1<br>NC2	Complete strongylid ITS-2	ACGTCTGGTTCAGGGTTGTT<br>TTAGTTTCTTTTCCTCCGCT	55	Species dependent

[1]Annealing temperature
[2]Amplicon length
